# An adaptive seamless phase II/III clinical trial design incorporating short-term endpoint information

**DOI:** 10.1186/1745-6215-12-S1-A2

**Published:** 2011-12-13

**Authors:** Nigel Stallard

**Affiliations:** 1 Statistics and Epidemiology, Division of Health Sciences, Warwick Medical School, University of Warwick, Coventry, CV4 7AL, UK

## 

Adaptive seamless phase II/III designs enable a clinical trial to be conducted in stages with the most promising of a number of experimental treatments selected on the basis of data observed in the first stage to continue along with the control treatment to the second and any subsequent stages. The main statistical challenge in such a design is ensuring control of the type I error rate. Most methodology for such trials is based on the same endpoint being used for interim and final analyses. In some settings the primary endpoint can be observed only after long-term follow-up. In this case it may be desirable to use short-term endpoint data along with any long-term data available at the interim analysis to inform treatment selection. If short-term data are available for some patients for whom the primary endpoint is not available, basing treatment selection on these data may lead to inflation of the type I error rate.

This talk presents a new method [1] that allows the use of short-term endpoint information for treatment selection whilst controlling the overall type I error rate. The method builds on the work of Galbraith and Marschner [2], who proposed a method for using short-term endpoints in interim analyses for comparison of a single experimental treatment with a control. The method is based on adjustment of the usual group-sequential boundaries [3,4] to allow for monitoring of a test statistic obtained from fitting a bivariate normal model to the correlated short-term and long-term endpoints. The model can be fitted either using a linear mixed model or by combining the results from separate linear regression models for the short and long-term data [5].

Result will be presented from a simulation study to investigate the properties of the new method. In addition to verifying control of the type I error rate, these results show that the use of the short-term endpoint data can lead to an increase in power when the short and long-term endpoints are correlated.

## References

[B1] Stallard N A confirmatory seamless phase II/III clinical trial design incorporating short-term endpoint information Statistics in Medicine 2010 29 959 971 20191605 10.1002/sim.3863

[B2] Galbraith S Marschner IC Interim analysis of continuous long-term endpoints in clinical trials with longitudinal outcomes Statistics in Medicine 2003 22 1787 1805 10.1002/sim.1311 12754715

[B3] Jennison C Turnbull BW Group-sequential analysis incorporating covariate information Journal of the American Statistical Association 1997 92 1330 1341 10.2307/2965403

[B4] Stallard N Todd S Sequential designs for phase III clinical trials incorporating treatment selection Statistics in Medicine 2003 22 689 703 10.1002/sim.1362 12587100

[B5] Engel B Walstra P Increasing precision or reducing expense in regression experiments by using information from a concomitant variable Biometrics 1991 47 13 20 10.2307/2532491

